# Clinical Characteristics, Diagnosis, and Treatment of Major Coronavirus Outbreaks

**DOI:** 10.3389/fmed.2020.581521

**Published:** 2020-11-13

**Authors:** Rupinder Mann, Abhilash Perisetti, Mahesh Gajendran, Zainab Gandhi, Chandraprakash Umapathy, Hemant Goyal

**Affiliations:** ^1^Department of Internal Medicine, Saint Agnes Medical Center, Fresno, CA, United States; ^2^Division of Gastroenterology and Hepatology, Department of Internal Medicine, University of Arkansas for Medical Sciences, Little Rock, AR, United States; ^3^Department of Internal Medicine, Paul L Foster School of Medicine, Texas Tech University, El Paso, TX, United States; ^4^Department of Medicine, Geisinger Community Medicine Center, Scranton, PA, United States; ^5^Division of Gastroenterology and Hepatology, Department of Internal Medicine, The University of Texas Health Science Center at San Antonio, San Antonio, TX, United States; ^6^Division of Gastroenterology and Hepatology, Department of Internal Medicine, The Wright Center of Graduate Medical Education, Scranton, PA, United States

**Keywords:** COVID-19, MERS, SARS, SARS-CoV-2, clinical

## Abstract

Human coronavirus infections have been known to cause mild respiratory illness. It changed in the last two decades as three global outbreaks by coronaviruses led to significant mortality and morbidity. SARS CoV-1 led to the first epidemic of the twenty first century due to coronavirus. SARS COV-1 infection had a broad array of symptoms with respiratory and gastrointestinal as most frequent. The last known case was reported in 2004. Middle East respiratory syndrome coronavirus (MERS-CoV) led to the second outbreak in 2012, and case fatality was much higher than SARS. MERS-CoV has a wide array of clinical presentations from mild, moderate to severe, and some patients end up with acute respiratory distress syndrome (ARDS). The third and recent outbreak by severe acute respiratory syndrome coronavirus-2 (SARS-CoV-2) started in December 2019, which lead to a global pandemic. Patients with SARS-CoV2 infection can be asymptomatic or have a range of symptoms with fever, cough, and shortness of breath being most common. Reverse transcriptase-Polymerase chain reaction (RT-PCR) is a diagnostic test of choice for SARS CoV-1, MERS-CoV, and SARS CoV-2 infections. This review aims to discuss epidemiological, clinical features, diagnosis, and management of human coronaviruses with a focus on SARS CoV-1, MERS-CoV, and SARS CoV-2.

## Introduction

Coronaviruses (CoV) are the largest group of viruses in Nidovirales order with spike-like projections, which led to the name “Coronavirus.” The CoVs have caused three global outbreaks in the last 20 years, with coronavirus disease-2019 (COVID-19) being the latest. The first epidemic of the twenty first century was Severe Acute Respiratory Syndrome (SARS) caused by SARS-CoV (SARS-CoV-1), which was first reported in November 2002 in Guangdong China, leading to 8,098 laboratory-confirmed cases with a case fatality rate of 9.6% globally (1, 2). The Middle East Respiratory Syndrome (MERS) caused by MERS-CoV was the second outbreak, first reported in Saudi Arabia in 2012 with 2,521 laboratory-confirmed cases with a case fatality rate of 36% (3). SARS-CoV-2 causes the third and most recent CoV outbreak (COVID-19). It first originated in Wuhan, China, after a cluster of patients presented with atypical pneumonia-like respiratory symptoms with a shared history of visits to a local Wuhan seafood market. Initially, the virus was thought to be a novel CoV and was labeled as 2019-novel CoV (2019 nCoV) (1, 4, 5). The outbreak was declared as a public health emergency by the World Health Organization (WHO) on Jan 30th, 2020 (6). It continued to spread globally and was declared a pandemic on March 11th, 2020, by WHO. The 2019-nCoV was later identified and renamed as SARS-CoV-2. SARS-CoV-2 is a zoonotic disease that most likely originated in bats. It primarily causes respiratory illness, very similar to SARS-CoV and MERS-CoV, with a much higher rate of transmission (7). The number of cases of COVID-19 continues to increase around the world, with more than 34.5 million cases and >1 million deaths worldwide as of October 2, 2020.

These outbreaks of SARS, MERS, and COVID-19 share many similarities, including the clinical presentation, transmission, and management. Although acute respiratory tract infections are the most common clinical manifestations, extrapulmonary symptoms are increasingly recognized (8–10). In a retrospective analysis of 138 SARS patients in Hong Kong, 28% of patients had watery diarrhea as their presenting complaint (11). In a meta-analysis based on COVID-19 patients, the pooled prevalence of gastrointestinal (GI) symptoms was found to be 17.6% (95% confidence interval [CI], 12.3–24.5%), and the RNA virus was detected in stool samples in about 48.1% (95% CI, 38.3–57.9%) of the patients (8). The case fatality of MERS (36%) is much higher than SARS (9.5%) and COVID-19 (2.3%) (3, 12).

SARS, MERS, and COVID-19 all have a zoonotic origin. Respiratory droplets also spread SARS infection. SARS was contained by public health measures like isolation of patients, tracing and strict quarantine of contacts, community quarantine, surveillance, and social distancing. The primary reservoir for MERS-CoV in dromedary camels. Although it is human to human transmission, most have the primary case started by acquiring infection from the camel. Most human to human transmission cases of MERS occurs while in close contact with infected persons like healthcare settings, households, and workplaces. Systematic and strict infection control measures in these situations have helped to limit the spread. Compared to SARS and MERS, COVID-19 is more transmissible but lower mortality, which led to wide transmission. Most cases are asymptotic to mild symptoms, and this, along with increased globalization since MERS and SARS infection, led to the spread of COVID-19 more rapidly. Based on lessons learned from SARS and MERS outbreaks, there is an increased international collaboration between various governments and organizations, which led to the rapid development of diagnostic tests after the Chinese Ministry of Health shared the genetic sequence SARS-CoV-2 virus.

This review aims to discuss the epidemiology, classification of CoV, clinical features, diagnosis, and management along with vaccine options for SARS, MERS, and COVID-19.

## Coronaviruses

**Figure 1:**
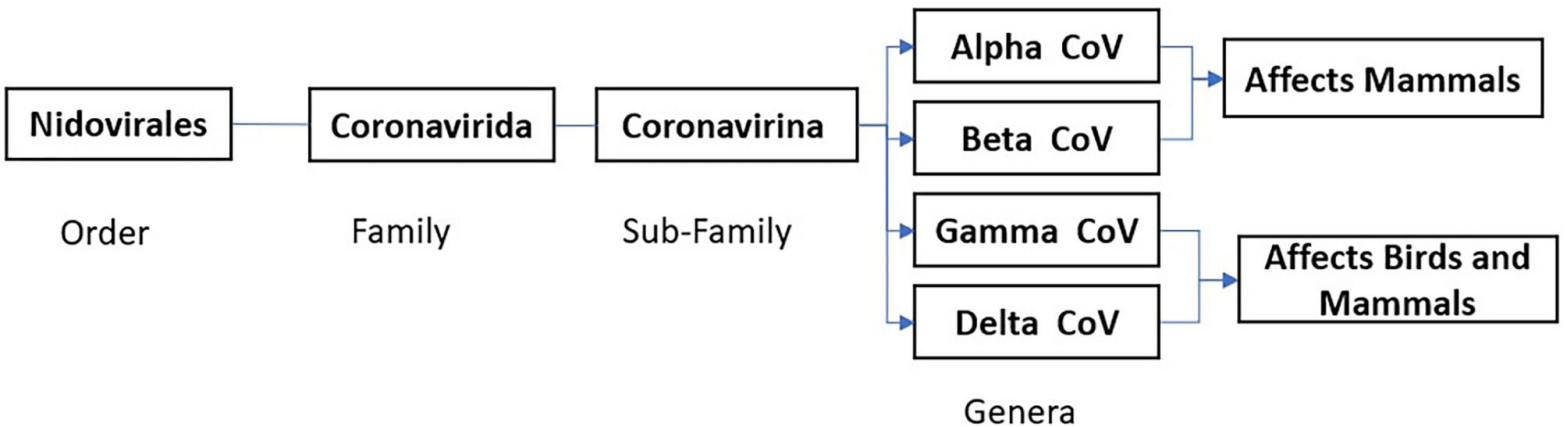
Corona Virus Classification based on International Committee Taxonomy of Virus (ICTV) ninth report 2011 (13).

The CoVs are RNA viruses of the Coronavirinae subfamily, Coronaviridae family, and Nidovirales order (International Committee of Taxonomy of Viruses) (Figure 1). Coronavirus is a group of large, single positive-sense, enveloped, highly diverse RNA viruses. The RNA genome is 27–32 kb in size, largest among RNA viruses, capped, and polyadenylated in nature (14–16). Under cryo-electron tomography and cryo-electron microscopy, CoV virions have a spherical shape around 125 nm in diameter, club-shaped spike projections arising from the virion's surface. These crown-like spikes give the appearance of a solar corona, thus naming them as coronavirus. The nucleocapsid is in the virion's envelope, and these nucleocapsids are helically symmetrical, which is not a common finding in positive-sense viruses (17).

The CoV genome has 6 to 10 open reading frames (ORFs). Spike (S) protein (trimeric), membrane (M) protein, envelope (E) protein and nucleocapsid (N) protein are structural proteins of CoV. Beta-CoVs also have hemagglutinin esterase (HE) glycoprotein. RNA has a cap structure at the 5' end and polyadenyl sequences at the 3' end. The 5' end codes for polymerase, followed by genes for envelope proteins and the nucleocapsid protein. The CoV genetic material is very susceptible to frequent mutations, leading to new strains of the virus with differing virulence (14, 18). Virions of CoV attach to the host cell surface receptors via its protein spikes and through the viral envelope's infusion with the plasma membrane of an endocytic vesicle releasing its genome into the host cell. The entire replication cycle occurs in the cytoplasm, involving the production of subgenome-sized (sg) minus-strand and full-length RNA intermediates. The viral genome serves as mRNA for the replicase polyproteins and a template for minus-strand synthesis (19).

### Coronavirus Classification

Coronavirinae is subdivided into four genera based on protein sequences, genomic structures, and phylogenetic relationships. Four genera are Alphacoronavirus (Alpha-CoV), Betacoronavirus (Beta-CoV), Gammacoronavirus (Gamma-CoV), and Deltacoronavirus (Delta-CoV) (15, 20). While Alpha-CoV and Beta-CoV are known to infect mammals, Gamma-CoV and Delta-CoV infect both birds and mammals. The primary host for Alpha-CoV and Beta-CoV are bats and rodents, while birds are the primary host for Gamma-CoV and Delta-CoV. Coronaviruses cause infections in avian and mammalian species manifesting in the form of respiratory illness (pneumonia, acute respiratory distress syndrome), GI symptoms (diarrhea, nausea, vomiting), hepatitis, encephalomyelitis, vasculitis, and coagulopathy. These viruses account for almost 30% of the common cold cases in human beings, mainly due to HCoVs (HCoV-OC43, HCoV-HKU1, HCoV-229E, and HCoV-NL63). The SARS, MERS, and COVID-19 can present with both respiratory and gastrointestinal symptoms (14, 18).

### Human Coronavirus (HCoV)

**Figure 2:**
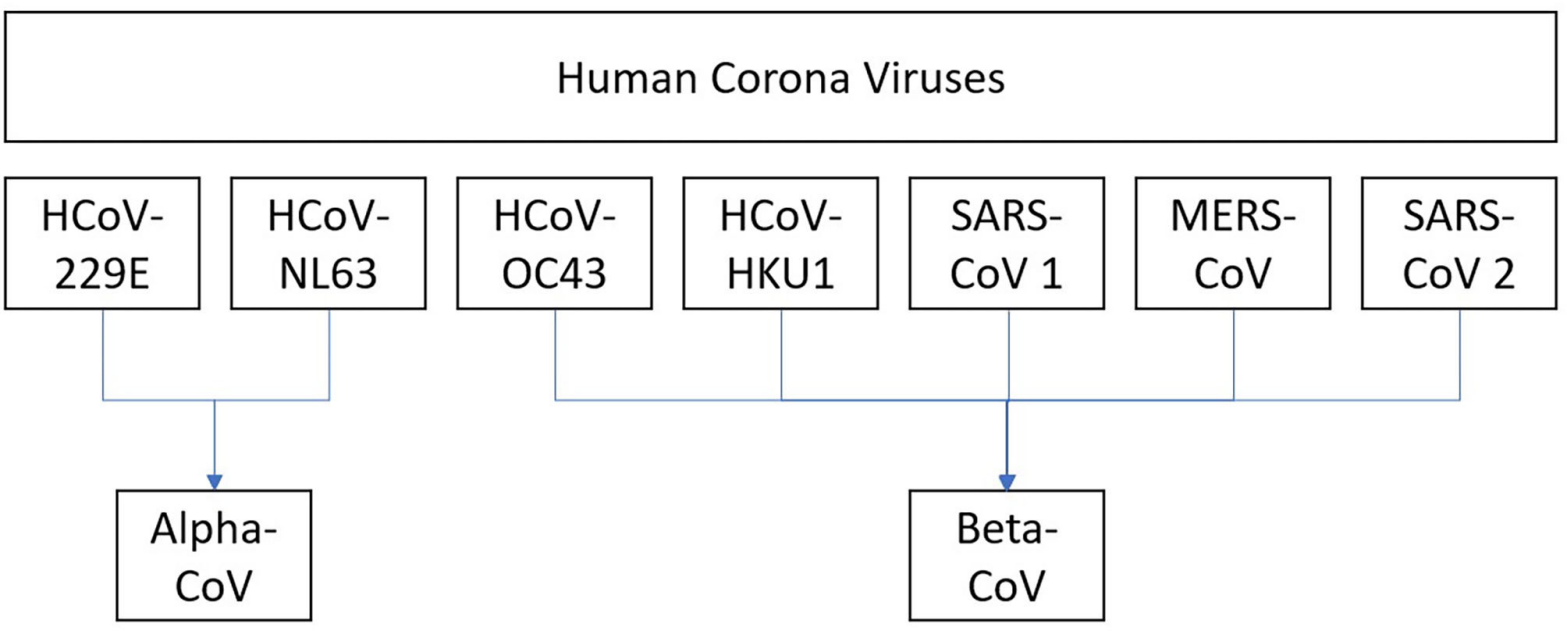
Human coronaviruses.

There are seven known HCoVs. All of these HCoVs have an animal origin and are found primarily in rodents or bats based on the current sequence databases (21). Out of seven, HCoV-229E and HCoV-NL63 are alpha-CoVs. HCoV-OC43, HCoV-HKU1, SARS-CoV-1, MERS-CoV, and SARS-CoV-2 are beta-CoVs (Figure 2) (7). SARS-CoV-1, SARS-CoV-2 MERS-CoV, HCoV-NL63, and HCoV-229E originated in bats, whereas HCoV-OC43 and HKU1 likely originated in rodents (20). The last three CoVs (SARS-CoV-1, MERS-CoV, and SARS-COV-2) have led to major outbreaks causing significant mortality and morbidity.

#### HCoV-229E

In 1966, HCoV-229E strain B814 was the first-ever isolated HCoV identified from the nasal passage of a patient who presented with the common cold. The patients infected with HCOV-229E present with symptoms of the common cold (sneezing, sore throat, headache, malaise, and 20–30% patients also have fever and cough). The incubation period is 2–5 days. HCoV-229E peaks during the winter season in tepid climates (7).

#### HCoV-OC43

HCoV-OC43 was first reported in 1967. While it has a similar clinical presentation, time of incubation, and epidemiology with HCoV-2294, but it has no serological cross-reactivity with HCoV-229E. The symptomatology due to these two viruses mimics those of influenza and rhinovirus. HCoV-OC43 has been shown to have infected neurons in *in-vivo* studies in mice and also neuroinvasive features clinically. It also peaks during the winter season in tepid climates (7, 22).

#### HCoV-NL63

The first case of HCoV-NL63 was reported from a 7 months-old girl in the Netherlands in 2004. Children under the age of 5 years are most commonly infected, but it can infect all age groups. The patient infected with HCoV-NL63 typically presents with coryza, fever, bronchiolitis, fever, and may even present with croup in some rare cases. The incubation period is typically 2–4 days. Patients with HCoV-NL63 have co-infection with other respiratory viruses in about 71% cases. It is globally widespread and peaks during early summer, spring, and winter seasons (7, 22).

#### HCoV-HKU1

HCoV-HKU1 was first discovered in 2004. HCoV-HKU1 presents as mild respiratory symptoms. It also peaks in the winter season, and the incubation period is 2–3 days (7). HCoV-229E, HCoV-OC43, HCoV-HKU1, and HCoV-NL63 are all transmitted by respiratory droplets and fomites. It accounts for up to 15–30% of respiratory infections in a year and causes more severe disease in the elderly, immunocompromised individuals (such as those with underlying co-morbidities and neonates) (17).

#### SARS-CoV

SARS-CoV or SARS-CoV-1 is the first coronavirus known to cause severe acute respiratory distress syndrome (ARDS). After the discovery of the SARS-CoV-2 virus in 2019, SARS-CoV is also referred to as SARS-CoV-1. SARS was first reported in 2002 and then spread globally with the last reported case in 2004. Infected patients presented with myalgias, malaise, fever, chills, cough, dyspnea, and respiratory distress as a late symptom. In severe cases, multi-organ involvement was reported (GI, liver, and kidney) (7). Diarrhea was reported in 40 to 70% of SARS-CoV-1 cases (9, 11, 23). Abnormal liver chemistries, elevated creatinine kinase, and lymphopenia were common laboratory findings. The route of transmission included respiratory droplets, fomites, and fecal-oral routes. The Chinese horseshoe bat was found to be a natural host of SARS-CoV-1 with the civet as an intermediate host. SARS-CoV-1 utilizes angiotensin-converting enzyme 2 (ACE2) receptors, which are almost omnipresent in the body (7, 17, 24).

#### MERS-CoV

MERS-CoV was first reported from Saudi Arabia in 2012. Patients present with fever, cough, chills, sore throat, myalgias, arthralgias, dyspnea, pneumonia, and acute renal failure. In up to 30% of patients, gastrointestinal symptoms like vomiting and diarrhea can be seen. The route of transmission is by respiratory droplets and fomites. Bats are likely the animal reservoir host, and dromedary camels are likely the intermediate host for human transmission. MERS-CoV utilizes Dipeptidyl peptidase 4 (DPP4) as its receptor (7, 17, 24).

#### SARS-CoV-2

Patients primarily present with fever, cough, and dyspnea. A systematic review and pooled analysis of 45 studies showed that fever (81.2%), cough (62.9%), loss of appetite (33.7%), shortness of breath (26.9%), loss of taste (25.4%), and sputum production (24.2%) were common symptoms reported by patients (25). Another systematic review and meta-analysis showed that fever (76.70%), cough (67.76%), olfactory (44.40%), gustatory (38.16%), dyspnea (37.49%), fatigue (29.93%), sputum production (17.85%), sore throat (16.7%), and headache (15.49%) were common symptoms observed in COVID-19 patients (26). The prevalence of gastrointestinal symptoms like diarrhea (9.1%), nausea/vomiting (5.2%), and abdominal pain (3.5%) were reported in COVID-19 positive patients (27). ARDS, acute respiratory failure, arrhythmias, septic shock, acute cardiac injury, cardiomyopathy, acute renal failure are common complications observed in these patients (25, 26). The primary transmission route is respiratory droplets, but there are reports of transmission via fomites or fecal-oral route have been seen (7, 21). SARS-CoV2 uses human ACE2 receptors, which is utilized by SARS-CoV-1, but it was found to have a higher affinity for these receptors than SARS-CoV-1, which in turn can partly explain why SARS-CoV-2 is more infectious than SARS-CoV-1 (28, 29).

## Severe Acute Respiratory Syndrome (SARS)

The first case of the severe acute respiratory syndrome (SARS) was found in Foshan city of Guangdong province in China on November 16th, 2002, and it spread to more than 30 countries across five continents. There has been a total of 8,098 cases and 774 deaths caused by SARS-CoV-1 (30, 31). WHO declared the end of the SARS epidemic in July 2003. Four more SARS-related incidents occurred from July 2003 to January 2004. Three of those incidents were due to laboratory biosafety breaches in Singapore, Taipei, and Beijing leading to the occurrence of seven cases. There were four sporadic community-acquired cases reported in China. No new cases of SARS have been reported since January 2004 (32). SARS-CoV-1 had a mortality rate of 9%, and mortality reached up to 50% in patients who were older than 60 years (33).

Multiple studies were performed to investigate the role of primary animal hosts and intermediary hosts as the outbreaks typically started in live animal markets in China. In a seroprevalence study conducted in Guangdong, China, 9.1% were tested positive for the SARS-CoV-1 IgG antibody. These positive IgG antibodies were higher in the animal trader group (13%) when compared to 1–3% of persons in control groups. Further investigation showed that these animal trader groups predominately traded “masked palm civets” among other animals (34). Another study showed that SARS-CoV-1 was isolated from other animals such as raccoon dogs and in humans working in the same market. All the animal isolates retained a 39-nucleotide sequence (35). Despite these findings, widespread SARS-CoV-1 infection was not noted in the civet cats suggesting that it was most likely an intermediate host (36). In 2005, one of the horseshoe bats species was found to have an 88–92% nucleotide sequence with SARS-CoV-1. This indicated that bats were more likely the natural host for this virus (37).

### Incubation Period

**Figure 3:**
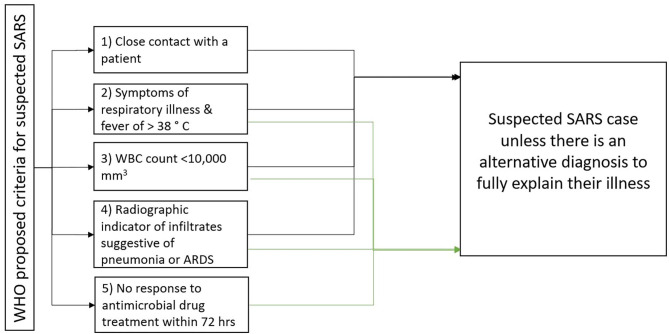
WHO criteria for suspected SARS case (36).

The estimated mean incubation period for SARS-CoV-1 infection was 4.6 days (95% Cl, 3.8–5.8 days), with 95% of cases having disease onset within 10 days, which could extend as long as 16 days (32, 36, 38). A study from Hong Kong on 1,755 patients showed that the average time from symptom onset to need for invasive mechanical ventilation and death was 11 and 23.7 days, respectively (38). The diagnosis is made by contact history, laboratory tests along with clinical manifestations (39). The WHO proposed five criteria to assist in the diagnosis, as depicted in Figure 3. Patients have suspected SARS if they meet criteria 1 to 4 (or) 2 to 5 unless they have an alternative diagnosis to explain their illness (36).

### Clinical Manifestations

Clinical symptoms of SARS include fever, chills, myalgia, malaise, dry cough, shortness of breath, and headache. Nausea, vomiting, dizziness, and upper respiratory symptoms like sore throat, rhinorrhea were less frequent (Table 1) (40, 41). In more than 60% of cases, radiographic changes were observed to be present on initial presentation, and in 41% of cases, the radiographic changes occurred before lower respiratory tract symptoms (39). Patients manifest symptoms in different stages. Fever, dry cough, myalgia, and malaise were presenting symptoms in the first week, which were shown to improve most patients. Returning of fever, along with worsening lung consolidation and respiratory failure, were observed during the second week in about 20% of the patients, which could potentially result in acute respiratory distress syndrome (ARDS) (32).

**Table 1:** Epidemiological and clinical features of SARS, MERS, and COVID-19.

**Disease**	**SARS**	**MERS**	**COVID-19**
First reported case [Year]	2002	2012	2019
Country of diagnosis	China	Saudi Arabia	China
Human Corona Virus [HCoV]	SARS-CoV-1	MERS-CoV	SARS-CoV2
Genera	Beta-CoV	Beta-CoV	Beta-CoV
Mode of transmission	Human to Human	Human to Human and Contact with infected camel	Human to Human
Natural reservoir	Bats	Bats	Bats
Intermediate host	Civet	Dromedary camels	Pangolins
Common clinical features	Fever, chills, malaise, dry cough, shortness of breath, headache, nausea, vomiting, diarrhea	Fever, chills, headache, runny nose, dry cough, sore throat, abdominal pain, nausea, vomiting, diarrhea	Cough, fever, shortness of breath, abdominal pain, diarrhea, vomiting
Laboratory findings	Marked lymphopenia, elevated ALT, elevated lactate dehydrogenase (LDH), pro-inflammatory cytokines	Leukopenia or lymphocytosis with lymphopenia, elevated transaminases, elevated LDH, elevated creatinine	Lymphopenia, elevated CRP, elevated AST, elevated procalcitonin level, elevated PT, aPTT, D-dimer, and ESR
Radiographic findings	Normal appearance, interstitial thickening, focal to multilobular airspace opacity with airspace opacities most common	Focal to multilobar airspace disease, ground-glass opacities, and occasional pleural effusions with ground-glass opacities being most common	Ground glass opacities (GGO), consolidation, paving stone sign, pleural thickening, vascular thickening, and fibrinous lesions common findings
Case fatality (%)	9.5	36	2.3
Number of cases and deaths	8,098 cases, 774 deaths	2,521 cases, 919 deaths (by Jan 16th 2020)	More than 8 million cases, 438,000 deaths (by June 16th, 2020)

Diarrhea was one of the common symptoms observed in patients with SARS (32, 39). In a retrospective study with confirmed SARS cases in Hong Kong, 28% of patients had watery diarrhea as presenting symptoms. Furthermore, 38.4% of patients developed diarrhea during illness. Diarrhea lasted for a mean duration of 3.7 days and resolved spontaneously in most cases. Moreover, SARS-CoV-1 RNA was detected in the stool for up to 10 weeks after the onset of symptoms (11). In children under the age of 12 years, symptoms were much milder than adults, but the teenage individuals had similar presentations as adults. Fortunately, there was no known mortality in young children and teenagers. The mortality rate increased with age, especially those with multiple comorbidities (32, 39). Elderly patients sometimes presented with atypical symptoms such as decreased well-being, confusion, and falls (32). Epidemiologic showed that asymptomatic infections were common in SARS. A meta-analysis showed that the overall seroprevalence among humans (except animal handlers) was 0.10% (95% Cl, 0.02–0.18). Healthy blood donors and individuals recruited from the health-care setting showed a seroprevalence of 0.16% (95% CI, 0–0.37) compared to overall prevalence (42). Furthermore, healthcare workers and individuals who had close contact with SARS patients had a higher seroprevalence of 0.23% (95% Cl, 0.02–0.45). Transmission of the virus occurred predominately after the fifth day of illness, probably due to low viral load in the upper respiratory tract (especially during the early phase of the illness). Unlike COVID-19, the lack of a large number of asymptomatic carriers and paucity of transmission in the early phase of illness (first 5 days) assisted in aggressive case detection, contact isolation, and control of this global outbreak (39) (Table 1).

### Diagnosis

#### Laboratory Diagnosis

Reverse transcriptase PCR (RT-PCR) assay for the detection of viral RNA is the test of choice for SARS diagnosis (Table 1). Viral RNA has been found in both upper and lower respiratory tract secretions, serum, stools, and urine specimens, enabling RT-PCR to be performed on all these samples (32, 39). As viral load is low during the first 5 days of illness, a negative specimen during this time does not exclude the diagnosis. Furthermore, the lower respiratory tract (sputum, tracheal aspirate, and bronchoalveolar lavage) samples have a higher viral load than those of the upper respiratory tract (nasal, pharyngeal, and nasopharyngeal). Therefore, a single specimen from the upper respiratory tract also does not rule out the diagnosis. Testing multiple specimens improves the rate of detection (39). Viral cultures could be used for diagnosis but takes a long time and require processing in biosafety level 3 facilities. Hence, they are restricted to special cases or for research purposes only (32, 39).

Marked lymphopenia involving both B and T lymphocytes (CD4 and CD8 subsets), and natural killer (NK) cells are observed in SARS patients (39). Low levels of CD4 and CD8 on presentation are associated with worse clinical outcomes (43). Pro-inflammatory cytokines and chemokines like interleukin 1 (IL-1), IL-6, IL-8, IL-12, C-C motif chemokine ligand 3 (CCL3), and CCCL10 levels also elevated (39). High Lactate dehydrogenase (LDH) level on admission is associated with higher mortality (38). Reactive hepatitis has been reported as a common complication in SARS patients. In a study of 294 SARS patients, 24% (70/294) had elevated alanine transaminase (ALT) on admission, and 69% (209/294) developed ALT elevation during the course of hospitalization (44). Liver function with elevated ALT increased further in patients who received systemic corticosteroid and ribavirin for treatment (32). Spontaneous recovery in the elevation of ALT was noticed in most patients with improvement in the disease. Though precise etiology for this abnormal ALT is unclear, cytokine release from inflammatory cells is the probable culprit (44). Other common laboratory abnormalities included acute kidney injury, elevated creatine kinase, and thrombocytopenia (45).

#### Radiographic Diagnosis

The common Chest X-ray findings are unilateral, or bilateral peribronchial thickening or airspace infiltrates (32, 46). High-resolution computer tomography (HRCT) can detect early lung parenchymal changes. Some of these include interlobular septal and intralobular interstitial thickening, consolidation, and ground-glass opacification, predominantly involving peripheral lung fields and lower lobes (32). While these findings are not pathognomonic, they are supplementary to the diagnosis of SARS patients.

### Treatment

#### Antiviral Therapy

##### Ribavirin

Ribavirin is a synthetic nucleoside analog that was used empirically for the treatment of the SARS patients during the outbreak in 2003. Clinical studies, including a retrospective case series, and one randomized clinical trial with multiple clinical arms, were performed to determine the effectiveness of ribavirin in SARS patients. However, no conclusive determination could be made (32, 47). In a study conducted in the Greater Toronto area with 144 patients, 126 patients were treated with a higher dose of ribavirin, about half the patients developed drop of hemoglobin (>2 g/dl), and 40% of patients had 1.5-fold increase transaminases (32, 46). Although the exact cause of the drop in hemoglobin is uncertain, the hemolysis was proposed to be the likely cause. Other adverse effects noticed with ribavirin included bradycardia and teratogenicity (48). There is no conclusive data that ribavirin was effective in SARS, and significant side effects were seen.

##### SARS-CoV protease inhibitors

Protease inhibitors block virus entry and/or inhibit protease (cathepsin L) lysis (49). A combination of Lopinavir and ribavirin showed clinically significant synergistic *in-vitro* activity against SARS-CoV-1 prototype HKU39849. It was used clinically in addition to a standard treatment protocol (50, 51). When compared with the standard treatment regimen (ribavirin and steroids) treatment, combination therapy with lopinavir and ribavirin showed a decrease in the overall mortality rate (15.6% vs. 2.3%, *P* < 0.05) and intubation rate (11% vs. 0%, *P* < 0.05) (51).

Other protease inhibitors like Nelfinavir, Calpain inhibitor VI (Val-Leu-CHO), and calpain inhibitor III (Z-Val-Phe-Ala-CHO) were studied *in-vitro* for potential effects in SARS (47). Nelfinavir is an HIV-1 protease inhibitor with a safety profile already established in humans, and it showed to inhibit the replication of SARS-CoV-1 in Vero E6 cells (52). Calpain inhibitor VI (Val-Leu-CHO) and calpain inhibitor III (Z-Val-Phe-Ala-CHO), which are cellular cysteine proteases, were found to be potent inhibitors for SARS-CoV in Vero Cell (53).

##### Viral binding inhibitors

The angiotensin-converting enzyme 2 (ACE2) is a cellular receptor that interacts with the S1 domain of the spike protein. Compounds and peptides that bind to ACE2 can be theoretically used as an agent for the treatment and prevention of SARS (47). Sui et al. showed that recombinant single-chain variable region fragments (scFvs) against the S1 domain of SARS spike protein could be used as a target to inhibit the virus. One such human monoclonal antibody includes 80 R, which can inhibit syncytia formation between ACE2 and spike protein. This agent has been studied *in-vivo* in animal studies to determine its clinical use for emergency prophylaxis and treatment of SARS (54).

##### Fusion inhibitors

*In-vitro* evidence shows that fusion inhibitors could be potentially used against SARS-CoV-1 as it prevents the attachment (fusion) of the viral envelope to the host cell membrane. Bosch et al. tested peptides derived from the membrane-proximal (HR2) and membrane-distal (HR1) (heptad repeat region) of the spike protein as inhibitors of SARS-CoV-1. HR2 but not HR1 peptides were found to be inhibitory against SARS-CoV-1 (55). Similarly, another *in-vitro* study showed that one peptide, CP-1 derived from the HR 2 region, inhibited SARS-CoV-1 infection at the molecular level (56). This inhibitory potency of the HR2 peptides against SARS-CoV-1 was initially promising, but none of them made it to the clinical trials.

#### RNA Interface

RNA interference treatment (RNAi) technology has been used to target human immunodeficiency virus (HIV), Hepatitis B, and Hepatitis C viral infections. It is a process by which small interfering RNA (siRNA) is administered, leading to mRNA degradation (47). In an *in vivo* study conducted by Zhang and colleagues, specific siRNAs targeting the S gene in SARS-CoV-1 were constructed, and it showed that siRNA could effectively and specifically inhibit gene expression of Spike protein in SARS-CoV-1 infected cells (57). SiRNA inhibitors were studied in 21 rhesus macaques, 20 of them in 5 groups (*n* = 4) infected with SARS-CoV-1 strain PUMCO1, and one individual was for observation (without infection). Five groups included two control groups (infection control, non-specific SiRNA control) and three treatment groups (prophylactic treatment, co-delivery, and post-exposure treatment). Over the next 20 days, they were observed for SARS-like symptoms, SARS-CoV-1 RNA presence, lung histopathology, and immunochemistry changes. Macaques in the treatment group had less severe SARS-like symptoms with the relief of fever, decreased viral levels, and lower acute diffuse alveolar damage. This study suggested that siRNA may be used to reduce the severity of disease and decrease viral load (58). Other compounds like glycyrrhizin, a component of liquorice root, nitric oxide, niclosamide (antihelmintic drug) have shown *in-vitro* activity against SARS-CoV-1 by inhibiting replication of the virus, and no clinical studies have been performed using these agents (47).

#### Steroids

Systemic steroids were administered as one of the mainstay therapy during the SARS outbreak. Although multiple reasons exist for their use, the primary mechanism appears to be the anti-inflammatory role of steroids. First, multiple patients affected with SARS show clinical features consistent with cryptogenic organizing pneumonia (COP), which respond to steroids and are likely caused by immune hyperactivity and cytokine dysregulation. Second, in patients with severe SARS, there was evidence of hemophagocytosis in the lung, attributed to cytokine dysregulation. Additionally, steroids might play a role in mitigating the clinical progression of pneumonia and respiratory failure association with a peak level of SARS-CoV-1 viral load mediated by the host inflammatory response (32, 47).

Steroids are used as adjunctive therapy to ribavirin treatment in most cases. If the patient's respiratory status deteriorated, pulse dose steroids were added in studies reporting improved clinical outcomes (47). Overall, data on the use of steroids is controversial and adverse events were noted. A retrospective cohort analysis showed that the use of pulse methylprednisolone was associated with an increased risk of 30 day mortality (adjusted odds ratio [aOR] 26.0; 95% CI, 4.4–154.8) (59). Furthermore, a systemic review concluded that systemic steroids were not associated with any definite benefits but had potentially adverse effects like infectious complications, avascular necrosis, and steroid-induced psychosis (60). Prolonged use of steroids can also increase the risk of nosocomial infections, such as disseminated fungal disease, metabolic derangements, psychosis, and osteonecrosis (32).

#### Interferon

Interferon-alfa (IFN-α) has been used in the treatment of Hepatitis B and C. A similar approach was tried in *in-vitro* studies against SARS-CoV-1 replication (47). Pegylated (PEG) IFN-α is shown to significantly reduce viral replication, excretion, and expression by type-1 pneumocytes when given prophylactically to macaques before experimental infection with SARS-CoV-1. Postexposure treatment with PEG IFN-α showed intermediate results only (61). In a study of 22 patients with SARS infection, patients who received IFN-alfacon-1 along with corticosteroid (combined approach) showed rapid resolution of radiographic lung abnormalities, lower levels of creatine kinase, rapid normalization of lactate dehydrogenase level, improved oxygen saturation (*p* = 0.02), and lower rates of tracheal intubation (11.1% vs. 23.1%) and death (0.0% vs. 7.7%) compared with the corticosteroid monotherapy group. When combination therapy was given during the late-stage to six critically ill patients, four died despite therapy. This suggests that treatment during the early stages of the disease is essential (62).

#### Convalescent Plasma

During the outbreak, one of the initially proposed hypotheses was to use convalescent plasma from a patient fully recovered from SARS to treat patients having active SARS infection (32, 47). A retrospective study comparing convalescent plasma and pulsed steroids showed that patients in the plasma group had a higher discharge rate (77% vs. 23%, *p* = 0.004) and lower mortality (0% vs. 23.8% *p* = 0.049) when compared to the steroid group (63). In another study, patients who received convalescent plasma before day 14 had a higher day 22 discharge rate than those who received after day 14 (58.3% vs. 15.6%; *P* < 0.001). Similarly, a higher discharge rate was observed in patients with PCR positive and seronegative for CoV at the time of plasma infusion compared to seropositive patients (66.7% vs. 20%; *P* = 0.001) (64). Monoclonal antibodies obtained from immortalized B-lymphocytes isolated from patients with SARS during the convalescence period were shown to neutralize virus infection *in-vitro* and prevent replication *in vivo* in the mouse model of SARS-CoV-1 infection (65). These studies implicated that convalescent plasma is more effective if given early during disease. It can be given during the early phase of SARS if there is another outbreak (51, 52).

### Prevention

#### Vaccines

Severe morbidity and mortality associated with SARS make it crucial to develop a safe and successful vaccine to prevent re-emergence and spread of disease (36). It is vital to develop protective immune responses, including neutralization antibody and cytotoxic T lymphocytes generation (66).

##### Inactivated vaccine

Inactivated vaccines consist of whole or a specific component derived from pathogen by killing or inactivating through various chemicals (formalin, β-propiolactone, and diethylpyrocarbonate) or radiation, which make the viral genome non-infectious while maintaining the structure of the virus and thus preserving antigenicity. Compared to a live vaccine, the inactivated vaccines are easy to prepare and cannot propagate disease in immunocompromised patients (67). Various studies on SARS-CoV-1 research showed that inactivated vaccines induce the production of neutralizing antibodies (68–70). The inactivated vaccine was administered to humans and was well-tolerated and elicited SARS-CoV-1 specific neutralizing antibodies (71). However, no data on vaccine efficacy is available due to a lack of a natural challenge (72).

##### Viral vector vaccines

In viral vector vaccines, vaccine antigen is produced *in situ* upon infections of cells. Vector virus can be either an attenuated virus or genetically alerted virus which cannot replicate (73). These vaccines have several features that make them induce efficiently both innate and B cell- and T-cell-mediated immune responses, including their ability to persist in the host as genetic material, ability to infect directly antigen-presenting cells. Adenovirus vectors have both spike and nucleocapsid proteins. Adenovirus vectors show variable results depending on the preparation, route of administration, and animal model used, but the challenge experiment has not been performed yet (67, 72).

##### Subunit vaccines

Subunit vaccines are comprised of purified antigen and only utilize antigenic components from the virus of interest. In the subunit vaccine, antigenic components are grown *in-vitro* and then harvested for vaccine use. This vaccine either contains a spike protein component or nucleocapsid protein. It induces a high level of B-cell and T-cell-mediated immune response and generates high titers of antibodies. However, there is no *in-vivo* experiment performed yet (67, 72).

##### DNA vaccines

DNA vaccines consist of plasmid DNA that code for viral antigen components, which are directly injected or otherwise inoculated in the vaccine. DNA vaccine induces both humoral and cellular immune responses. It also uses spike peptides to induce high titers of neutralizing antibodies. Although DNA vaccines have shown promise in preclinical models, their success in the clinical studies has been unsatisfactory (67, 72).

##### Live attenuated vaccines

These vaccines are made by decreasing or removing the virulence of live virus by using chemical or site-directed mutagenesis. This process makes the virus an attenuated pathogen capable of producing a subclinical infection. The live vaccine will result in an innate and adaptive immune response, which can last life-long. The efficacy and immunogenicity of a live attenuated vaccine consisting of a recombinant SARS-CoV-1 lacking E gene were studied (67, 72). In a study, Hamsters immunized with recombinant SARS-CoV-1 without E gene developed a high level of serum-neutralizing antibody titers, and they were protected from replication of homologous (SARS-CoV Urbani) and heterologous (GD03) SARS-CoV-1 in both upper and lower respiratory tract (74). Thus, the deletion of a gene may be the first step toward developing a live attenuated SARS-CoV-1 vaccine (72).

## Middle Eastern Respiratory Syndrome

### Epidemiology

MERS-CoV was first isolated from the sputum of a 60 year male from the city of Jeddah in Saudi Arabia on September 20th, 2012. A pancoronavirus RTPCR assay was used to isolate this virus (75). This patient died due to renal failure and severe respiratory disease due to MERS-CoV (76). MERS became an epidemic with 2521 laboratory-confirmed cases and 919 deaths (case fatality rate 36%) (3). MERS-CoV cases are predominately reported from the Arabian Peninsula, with around 84% from Saudi Arabia (3). Twenty-seven countries have reported cases of MERS. All cases outside the Arabian Peninsula had either history of travel to the region or contact with someone who traveled to the region (3, 77).

The primary host of MERS-CoV remains unknown, and there is no definitive epidemiologic evidence linking MERS-CoV infection and bats. When more than 1,000 samples from *Taphozous perforates* bats (also called Egyptian tomb bat, species of Emballonuridae family) were analyzed, only a small amount of MERS-CoV closely matching to a human MERS-CoV was found (77). Dromedary camels (*Camelus dromedarius)* are major reservoir/intermediate hosts for MERS-CoV. Although there are cases of human-to-human transition, especially in health care settings due to close contact, while delivering unprotected care to a patient, the virus does not pass easily from the human-to-human (78). The WHO data shows that men are being affected more compared to women. The 50–59 years and 30–39 years age groups are at the highest risk of acquiring infection of primary and secondary cases, respectively (79).

### Incubation Period

The median incubation period is estimated to be around 5.2 days, ranging from 1.9 to 14.7 days. The time interval between symptom onset in a patient and symptoms in contact was about 7.6 days (95% CI, 2.5 to 23.1) (80). Approximately 4 days is the median time from illness onset to hospitalization with a median length of stay of 41 days (76). The incubation period was also found to be correlated with the severity of the disease. The mean incubation period was shorter for patients who died compared to those who survived (81).

### Clinical Manifestations

#### Pulmonary Symptoms

MERS has no specific signs and symptoms but mainly presents with respiratory manifestations. Clinical presentation ranges from asymptomatic cases to mild, moderate, severe disease with ARDS, multi-organ failure, and death (76, 77). These patients initially present with mild symptoms of low-grade fever, chills, headache, runny nose, dry cough, sore throat, dyspnea, and myalgia (Table 1) (76, 77). Patients can also have other respiratory tract symptoms like sputum production, wheezing, chest pain, headache, and malaise (80). Patients can deteriorate rapidly with progression to ARDS within a few days (80, 82, 83). Severe cases can present with pneumonia, ARDS, encephalitis, myocarditis, acute renal failure, secondary bacterial infection, or other life-threatening complications (83, 84).

#### Extrapulmonary Symptoms

Various extrapulmonary manifestations have been reported in patients with MERS, including acute renal impairment, which was present in up to half of patients. About 1/3rd of severely ill patients have GI symptoms. Anorexia, abdominal pain, nausea, vomiting, and diarrhea are common GI symptoms seen in patients with MERS (76, 77, 82). Other extrapulmonary manifestations include neurological, cardiac manifestations, hepatic and hematological complications. Cardiac complication includes pericarditis, arrhythmias, and hypotension. Neurological complications like ataxia, confusion, coma, and focal neurological symptoms were seen in a retrospective study of three patients in ICU from Saudi Arabia (85). In a single-center retrospective study of 70 patients, the majority of patients were old with a median age of 62 years, and 95.7% of patients with confirmed MERS-CoV infections were symptomatic. Studies also found arrhythmias in 15.7%, disseminated intravascular coagulation (DIC) in 14.7%, liver dysfunction in 31.4%, and acute kidney injury in 42.9% of the patients (86).

#### Risk Factors

Risk factors associated with severe MERS include old age, male gender, existing co-morbid conditions, low serum albumin, superimposed bacterial infections, and weaker immune system. About 76% of patients with MERS reported having at least one underlying co-morbid condition. The most common co-morbid conditions seen in hospitalized MERS patients were obesity, diabetes, hypertension, cardiovascular diseases, or end-stage renal disease, and these chronic diseases are thought to attenuate innate immunity response by down-regulating production of pro-inflammatory cytokines such as interferon-gamma (IFN-g) and interleukins (ILs) (76, 77, 84). The patients who died had increased frequency of comorbid conditions when compared with recovered or asymptomatic cases (86.8% vs. 42.4%, *p* < 0.001). The most commonly reported co-morbid condition included chronic renal failure (13.3%), diabetes (10.0%), and heart disease (7.5%) (87). Lungs of smoker patients have shown upregulation of DPP4 receptors, making them more prone to have severe disease than a non-smoker (77).

### Diagnosis

No specific clinical features or radiographic features differentiate MERS from other respiratory viral infections, and diagnosis relies on laboratory findings (Table 2).

**Table 2:** WHO released the last update for case definition (confirmed and probable case) for classification and reporting purposes on July 26th, 2017 (88).

**Updated case definition by WHO July 26th, 2017**	
Confirmed case	1. Patient with laboratory-confirmed MERS, regardless of clinical signs and symptoms
Probable cases	1. Patient with febrile acute respiratory illness with clinical, radiological, or histopathological evidence of pulmonary parenchymal disease, and a direct epidemiologic link with case of laboratory-confirmed MERS case; and laboratory testing for MERS-CoV is unavailable, negative on a single inadequate specimen or inconclusive
	2. Patient with febrile acute respiratory illness with clinical, radiological, or histopathological evidence of pulmonary parenchymal disease that cannot be explained entirely by any other etiology; and patient resides or traveled to the Middle East or another country where MERS-CoV is known to be circulating in dromedary camels or where human infections have recently occurred; and laboratory testing for MERS-CoV is inconclusive
	3. Patient with an acute febrile respiratory illness of any severity; and has a direct epidemiologic link with a confirmed MERS-CoV case, and laboratory testing for MERS-CoV is inconclusive

#### Laboratory Diagnosis

Real-time reverse-transcription polymerase chain reaction (rRT-PCR) is a diagnostic test that is widely used for MERS infection as it is highly sensitive with a short turnaround time (Table 3) (77, 80). Three rRT-PCR assays are developed and routinely used for the detection of MERS-CoV. Assays target upstream of the E protein gene (UpE), the open reading frame 1b (ORF 1b), and 1a (ORF 1a). The assays for the UpE and ORF-1a targets have 100% sensitivity (95% CI, 91.1–100%) in detecting the infection (90). UpE assay is recommended for screening and ORF-1a or ORF-1b assay for confirmation (89).

**Table 3:** WHO interim guidance, Jan 2018: MERS-CoV Detection by NAAT/PCR (89).

**MERS diagnosis based on nucleic acid amplification test** (**NAAT) testing**	
Laboratory confirmed case	Two positive NAAT assays with different targets/sequencing on the MERS-CoV genome or One positive NAAT result for a specific target on the MERS-CoV genome and MERS-CoV sequence confirmation from a separate viral genomic target
Probable	Patients with a positive NAAT result for a single specific target without further testing but with a history of potential exposure and consistent clinical signs with MERS

Sample can be collected from upper respiratory tract specimens (nasopharyngeal and oropharyngeal) and lower respiratory tract specimens (sputum, tracheal aspirate, or lavage). Lower respiratory tract specimens have higher viral load than upper respiratory tract specimens as Dipeptidyl peptidase 4 (DPP4) receptors are expressed on non-ciliated bronchial epithelial cells and alveolar epithelial cells but not in upper respiratory tract epithelium. DPP4 are cellular receptors for MERS-CoV. Swabs from nasopharyngeal and oropharyngeal specimens should be collected on kits, which contain viral transport medium and both swabs from nasopharyngeal and oropharyngeal specimen should be placed in the same tube to increase the viral load (89).

If the first test, particularly upper respiratory tract specimen, comes negative in a patient with suspected MERS, a repeat test should be done, especially from lower respiratory tract specimens. In order to confirm the clearance of the virus, respiratory samples should be tested until there are two consecutive negative samples, and samples should be taken at least 2–4 days apart (89) (Table 3).

The infectious MERS-CoV virus can also be isolated from blood, urine, and fecal sample by culture but takes longer than RT-PCR (76, 80). MERS-CoV has also been isolated from environmental objects such as bedsheets, bedrails, intravenous fluid hangers, and X-ray devices in healthcare settings (76, 80). For antibody detection, paired serum samples are needed for the confirmation of infection. A single sample can provide information regarding prior infections or identifying probable cases, provided that the sample was taken at least 21 days after onset of illness. For paired samples, the first sample should be collected during the first week of illness, and two samples should be collected 3–4 weeks apart. Viral cultures are not recommended as a routine diagnostic test (89). Furthermore, viral culture and antibody detection assay using the whole virus should be done in specific laboratories that are biosafety level 3 (BSL-3) laboratories in the WHO Laboratory Biosafety Manual (80, 89).

Similar to SARS, laboratory abnormalities in MERS include leukopenia, thrombocytopenia, and elevated transaminases, lactate dehydrogenase, and creatinine levels. These are non-specific and can be found in other coronaviruses. Occasionally anemia, creatine kinase, C-reactive protein, and procalcitonin elevation, and hyponatremia are noted (76, 77, 80).

#### Radiologic Diagnosis

Abnormal chest radiograph findings are found to be more common in patients with MERS (90–100%) than with SARS (60–100%) (91). Airspace opacity was the most common abnormality in SARS patients, whereas ground-glass opacities were found more commonly in MERS patients (45). Chest X-ray findings are non-specific and similar to various viral pneumonia associated with ARDS. In severely ill MERS patients, chest radiograph and computed tomographic (CT) scan showed abnormalities in almost all patients, and it ranges from a mild unilateral focal lesion, bilateral multilobar airspace disease, ground-glass opacities, and occasional pleural effusions (76, 80). Thoracic imaging is usually normal in mild cases. The most common features seen on thoracic CT scans are bilateral, predominantly basilar, and subpleural air space involvement, with extensive ground-glass opacities and pleural effusions. Thoracic CT imaging done 3 weeks after onset of symptoms could reveal fibrotic changes, traction bronchiectasis, and architectural distortion (80, 82).

### Treatment

The treatment is mostly supportive with the goal of reducing the risk of complications like a secondary bacterial or viral infection, respiratory failure, and multiorgan failures in MERS. Supportive care includes rest, intravenous fluids, analgesics, and also broad-spectrum antimicrobial, antivirals, and antifungals to minimize the risk of co-infection with opportunistic pathogens if needed. Other supportive care is based on organ dysfunction and management of complications like using a ventilator for patients with respiratory failure (76, 77).

Although there are some treatments available, they are not specific to treat MERS-CoV (77).

#### Antibiotics

Broad-spectrum antibiotics are commonly given empirically during the management of MERS to treat bacterial pneumonia. A retrospective study of 93 patients reports 23.6% bacterial infection in patients with MERS, Legionella *pneumophila*, and Streptococcus *pneumoniae* are the most common agents, and so broad-spectrum antimicrobial should be considered for MERS patients (92). In critically ill patients, macrolide therapy was not associated with a difference in clearance of MERS-CoV RNA and improvement in 90 day mortality (93). Teicoplanin is a glycopeptide antibiotic isolated from Actinoplanes teichomyceticus and known to be active against gram-positive bacterial infections. *In-vitro*, it has been shown to inhibit the entry of MERS-CoV pseudotyped viruses into host cellular cytoplasm. There are no pharmacodynamic studies of this antibiotic specific to MERS-CoV, which are required to understand its antiviral efficacy (94, 95).

#### Antivirals

##### Ribavirin

Ribavirin is a nucleoside analog activated to a nucleotide by host kinases. Ribavirin was shown to inhibit MERS-CoV replications *in-vitro* (vero cells), but the dose is too high to be achieved *in vivo*. The 50% inhibitory concentration (IC50) of ribavirin was 41.45 microgram/ml, whereas a 1,000 mg intravenous dose of ribavirin can only achieve a level of up to 24 microgram/ml in human beings (95, 96). Ribavirin and interferon combinations inhibit MERS-CoV replication *in-vitro*. When used in combination, the required dose for IFN-α2b and ribavirin decreased by 8- and 16-folds, respectively. The combination also was shown to improve clinical outcomes in non-human primates (rhesus macaques and common marmoset) infected with MERS-CoV within 8 h of virus inoculation (76, 95). When this combination was tested in a severely ill patient, it showed improvement in survival at 14 days but not at 28 days, which was most likely due to administration in the advanced stages of the disease (97). A retrospective cohort study looked at a combination of ribavirin with IFN-α2a or IFN-β1a to treat MERS-CoV infection. Mortality rate was 85% vs. 64% (*p* = 0.24) in IFN-α2a and IFN-β1a, respectively (98). Although most of the data is available from small studies, a combination of ribavirin and interferon may be considered in MERS patients, especially in the early stages of the disease.

##### Protease inhibitors

Protease inhibitors are a well-known anti-retroviral agent, being used in the treatment of HIV. Lopinavir and Nelfinavir were shown to inhibit MERS-CoV *in-vitro*. Mean 50% effective concentration (EC50) of lopinavir using Vero E6 and Huh7 cells was 8.0 μM (96). An ongoing randomized controlled trial comparing the efficacy of treatment with a combination of lopinavir/ritonavir and recombinant IFN-β1b provided with standard supportive care with placebo and standard supportive care treatment in patients with laboratory-confirmed MERS requiring hospitalization (99).

#### Mycophenolic Acid

Mycophenolic acid (MPA) is an inhibitor of cellular inosine monophosphate dehydrogenase and inhibits purine synthesis in lymphocytes. In an *in-vitro* study, MPA showed strong inhibition of MERS-CoV with an IC50 of 2.87 μM. Similarly, IFN-β showed the most robust inhibition of MERS-CoV *in vitro*, with an IC50 of 1.37 U ml-1 compared to other interferon products (IFN-a2b, IFN-c, IFN-universal, IFN-a2a, and IFN-b). IFNβ, MPA alone, or in combination may be a useful post-exposure intervention in high-risk patients with known exposures to MERS-CoV or treatment of MERS-CoV (100). In a retrospective chart review study involving 51 patients, patients with MERS-CoV infection received different treatments, including broad-spectrum antibiotics, steroids, various antivirals, and mycophenolate mofetil. Eight patients who received mycophenolate mofetil and IFN-β survived, but this group of patients had low lower Acute Physiology and Chronic Health Evaluation II (APACHE-II) scores compared to other groups (101).

#### Resveratrol

Resveratrol has shown antiviral properties against many human viruses like the influenza virus, Epstein–Barr virus, herpes simplex virus, respiratory syncytial virus. Antiviral effects of resveratrol against MERS-CoV observed *in-vitro* due to observed inhibition of MERS-CoV nucleocapsid (N) protein expression. It can also prolong cellular survival due to the downregulation of apoptosis induced by MERS-CoV. However, there are adverse effects also reported with resveratrol like increasing viral RNA replication during Hep-C virus infection *in-vitro* (OR6 cells), and potent cytotoxicity in cultured cells. This drug needs to be studied further for its antiviral properties, with careful consideration to be given for potential adverse events (76).

#### Fusion Inhibitors

Fusion inhibitors are antiviral peptides, which prevents MERS-CoV entry into host cells by targeting various S protein areas. Camostat, a serine protease inhibitor and the heptad repeat 2 peptide (HR2P), a synthesized peptide are two MERS-CoV fusion inhibitors that were tested *in vitro*. Camostat suppressed MER-CoV viral entry into human bronchial submucosal gland-derived Calu-3 cells by 10-fold but was not efficacious against the immature lung tissue. HR2 blocks MERS-CoV replication and the spike protein-mediated cell-cell fusion (95, 96). Although fusion inhibitors have shown effects *in vitro*, and no *in vivo* clinical data available.

#### Interferon

*In vitro*, IFN-β has higher antiviral activity on MERS-CoV when compared to SARS-CoV (102). ORF4a inhibits IFN-β production through inhibitions of interferon regulatory transcription (IRF-3) factors and nuclear factor (NF)-κB actions (103). Among *in-vitro* studies, IFN-β is more potent that IFN-α2b, IFN-α2a, IFN-γ, IFN-universal type 1 with IC50 of 1.37 U/ml (96). Animal and *in-vitro* studies showed that IFNs have synergistic effects when used in combination with ribavirin, mycophenolate, which is discussed above in the mycophenolate and ribavirin sections.

#### Corticosteroids

High-dose systemic corticosteroids were given to treat many patients with severe MERS-CoV disease with the intention to reverse the progression of respiratory distress and to prevent lung fibrosis but turned out to be futile (87). A multicenter retrospective study of 309 critically ill ICU patients with MERS-CoV infection showed that patients who got corticosteroids were more likely to be on a ventilator (93.4% vs. 76.6%, *P* < 0.05) compared to patients who did not receive steroids. After adjusting for time-varying confounders, corticosteroid therapy was not significantly associated with 90 day mortality (aOR 0.75; 95% CI, 0.52–1.07) but was associated with delayed MERS-CoV RNA clearance (adjusted hazard ratio [HR], 0.35; 95% CI, 0.17–0.72; *P* = 0.005) (104). Steroids should be avoided in patients with MERS unless they are indicated for other clinical conditions as their safety is not clear in patients with MERS-CoV (82).

#### Convalescent Plasma

Convalescent plasma therapy involves the use of plasma or whole blood from patients with MERS-CoV infection who recovered fully from the disease. During the MERS outbreak in Korea in 2015, 3 of 13 patients with MERS infection with respiratory failure received four convalescent plasma infusion from recovered MERS patients. However, only two of four donor plasma showed neutralizing activity; therefore, the donor plasma should be tested for neutralizing activity. Only the donor plasma with a plaque reduction neutralization test (PRNT) titer 1:80 showed meaningful serologic effects after convalescent plasma infusion. ELISA IgG can be used as a substitute for neutralization tests in limited resource situations as it can predict PRNT titer ≥1:80 with >95% sensitivity and 100 % specificity with OR of 1.6 and 1.9, respectively (105).

#### Monoclonal Antibodies (mAbs)

Monoclonal antibodies are commonly used in various diseases, including infectious diseases. Mersmab1, first developed by Du et al., binds to the MERS-CoV spike protein receptor-binding domain (RBD) and thus competitively blocks the binding of the RBD to its cellular receptor, DPP4 (106). Three human monoclonal antibodies m336, m337, and m338 were identified from a large naïve-antibody library, and these antibodies target the receptor (CD26/DPP4) binding domain (RBD) of the MERS-CoV spike glycoprotein. All three human monoclonal antibodies have neutralizing activity and highest with m336 (107). Given the above results, mAbs can be developed as one of the treatment options against MERS-CoV in humans. A phase 1 randomized, double-blinded, placebo-controlled, first-in-human trial has been performed to study the safety, tolerability, pharmacokinetics, and immunogenicity of single ascending doses of a co-administered REGN3048 and REGN3051 monoclonal antibody but results have not been published yet (108).

Multiple other drugs like chloroquine, chlorpromazine, loperamide, Nitazoxanide, and cyclosporin, have also shown activity against MERS-CoV *in-vitro* but no *in-vivo* studies are available (95, 96).

### Prevention

#### Vaccines

For the development of vaccines against MERS-CoV, viral enveloped protruding spike (S) glycoprotein and its RBD and/or the nucleocapsid (N) protein are primary targets (109, 110). Various vaccines are under development, and it includes subunit, DNA, recombinant vector, and live attenuated vaccines.

##### Subunit vaccines

Protein subunit vaccine has defined one or more immunogenic components, and subunit antigen induces antibody responses with primarily CD4 T-cell responses. These vaccines have low risk *in vivo* compared to other vaccine types and are generally well-tolerated (110, 111). A recombinant protein containing residues 377–588 in the truncated receptor-binding domain of MERS-CoV spike (S) protein was fused with human IgG Fc fragment (S377-588-Fc) in an *in-vitro* culture of transfected 293T cells. In vaccinated mice, recombinant S377-588-Fc induced strong MERS-CoV S-specific antibodies, which blocks binding of RBS to DPP4 receptors and thus inhibits MERS-CoV infection. It shows that truncated RBD can be a potential candidate for a future safe vaccine against MERS-CoV (112).

##### DNA vaccines

DNA vaccines are safe, yield stable antigen expression, and cause only low-grade adverse effects like local pain at the injection site, and malaise or fever (110, 111). Although DNA vaccines induce lower immune response compared to other vaccines type, it induced both humoral and cellular immune response at low cost than others (111). Phase 1 open-label clinical study of GLS-5300 MERS-CoV DNA vaccine was conducted, and 75 healthy adults aged 18–50 years were enrolled in this study. These individuals were divided into three groups of 25, and each group received different doses (0.67, 2, or 6 mg) of the vaccine. The most common adverse effect in all groups was the injection site reaction (93%). As measured by S1-ELISA, seroconversion occurred in 66, 86, and 94% participants after first, two, and three vaccination, respectively. Neutralizing antibodies against MERS-CoV EMC-2012 infection of Vero cells were seen in 43, 39, and 3% at week 14, week 24, and at the end of the study, respectively. The B-cell and T-cell responses were 77 and 64%, respectively, at week 60. This vaccine should be tested further in MERS endemic area for efficacy (113).

##### Vector vaccine

Vector vaccines ChAdOx1 MERS, replication-deficient simian adenovirus vector (ChAdOx1), and modified vaccinia virus Ankara (MVA) based vaccine is known as MVA-MERS-S and already went through phase 1 clinical trial. Phase 1 open-labeled, non-randomized, uncontrolled trial for ChAdOx1 MERS was conducted between March 14 and August 2018 at Oxford, UK. Twenty-four healthy people aged 18–50 years with negative pre-vaccination tests for HIV antibodies, hepatitis B surface antigen, and hepatitis C antibodies received a single intramuscular injection of ChAdOx1 MERS at three different doses (5 × 10^9^ viral particles, 2.5 × 10^10^ viral particles, and 5 × 10^10^ viral particles for low, intermediate and high dose group, respectively). No serious adverse effects were reported in all three groups with different doses during 12 months follow-up. Seroconversion was 75, 92, and 68%, respectively in all groups at 14, 56 days, and 1 year after vaccination. From baseline, a significant increase in both T-cell (*p* < 0.003) and IgG (*p* < 0.0001) to the MERS-CoV spike antigen was seen at all doses. These results support the clinical development progression of phase 1b and 2 trials, especially in the endemic area (114).

In Germany, an open-label phase 1 clinical trial was done for the MVA-MERS-S vaccine, and this trial included healthy aged 18–55 years individuals with no clinically significant health problems with key exclusion criteria of previous MVA vaccination. Individuals were allocated to two different doses groups as one being the low-dose group (1 × 107 plaque-forming unit p) and the other being the high-dose group (1 × 108 PFU). These individuals received two doses of vaccine 28 days apart via the intramuscular route. No severe or serious adverse effects were noted. After the second dose of vaccine, seroconversion using a MERS-CoV S1 ELISA at any timepoint during the study was found to be 75% in the low dose group and 100 in the high-dose group. MERS-CoV spike-specific T-cell responses were detected in 83 and 91% of participants in the low-dose and high-dose group, respectively (115).

##### Live attenuated vaccine

Live attenuated vaccines can induce a potent immune response as they present antigens to the host immune system similar to natural infection. In animal models, a live attenuated vaccine for MERS-CoV has shown efficacy (110, 111). An engineered mutant virus lacking structural E protein, rMERS-CoV-ΔE genome replicated after cDNA clone was transfected into cells and was only efficiently disseminated in cells expressing the E protein in trans. The rMERS-CoV-ΔE mutant virus can be a potential vaccine candidate for MERS-CoV (116). Live attenuated vaccine CoV accessory proteins, and nsp16-deficient MERS-CoV vaccine have also been considered (110).

## Coronavirus Disease 2019 (COVID-19)

The first cases of COVID-19 were reported from Wuhan, China. In December 2019, cases of pneumonia of unknown cause occurred in Wuhan, Hubei Province of China, who had exposure to animals sold in the local Hunan seafood market (117–119). On January 7th, 2020, a new CoV type was isolated from these patients with pneumonia. Within a few days, the genetic sequence of this novel CoV (SARS-CoV-2) was identified (120). On January 30th, 2020, WHO declared the SARS-COV-2 outbreak as a Public Health Emergency of International Concern (PHEIC), and on March 11th, 2020, COVID-19 was declared as a global pandemic (121).

Human-to-human transmission due to close contact further caused the spread of the virus to other provinces during the Spring festival season in China. Within a span of a few weeks, It spread globally to multiple nations throughout the World (122). The first case outside China was reported on Jan 13th, 2020, in Thailand. As of July 4th, 2020, there are more than 11 million cases and 530,000 deaths worldwide. As of today, the United States (US) has the maximum number of cases followed by Europe (123). Human-to-Human transmission occurs due to direct contact or through respiratory droplets spread by coughing/sneezing or directly through fomites (124). SARS-CoV-2 can be detected in respiratory secretion up to 2 weeks after disease symptoms resolve. A study of 73 patients from china showed that 54.3% of patients were found to have positive SARS-CoV-2 RNA in the stool samples (125).

More than 75% of CoV infections have animals as a source of infection, and bats are considered as a reservoir for all human coronaviruses. There is still uncertainty about the intermediate host that led to human transmission (122). Pangolins are considered as a probable intermediate host for SARS-CoV-2 as Pangolin-CoV is 91 and 90.55% identical to SARS-CoV-2 and BatCoV RaTG13, respectively. BatCoV RaTG13 from *rhinolophus affinis* shares a 96% whole-genome identity with SARS-CoV-2 (126).

### Incubation Period

In a study of initial cases from Wuhan, China, the median age of these patients was 59 years, ranging from 15 to 89 years. The mean incubation period was estimated to be 5.2 days (95% CI, 4.1–7.0) (127). A study of publicly reported cases outside Hubei province found a median incubation period of 5.1 days (95% Cl, 4.5–5.8), and symptom onset was within 11.5 days (95% Cl, 8.2–15.6 days) in 97.5% of infected patients (128). Given the above information, 14 days quarantine or medical observation will identify an active case in more than 97% of exposed patients. These studies have limitations; they included mostly hospitalized patients who can confound results, as the incubation period may differ in mild cases.

### Clinical Features

Patients with SARS-CoV-2 infection can be asymptomatic or have a wide range of symptoms (Table 1). Mild cases are reported to recover within 1 week, and severe cases developed progressive respiratory failure leading to death (118). In a prospective study of 16,749 patients with COVID-19, cough (70%), fever (69%), and shortness of breath (65%) were the most common symptoms. Almost 29% of patients presented with enteric symptoms along with respiratory symptoms, and only 4% have just enteric symptoms alone (129).

A meta-analysis of 47 studies showed pooled prevalence of diarrhea, nausea/vomiting and abdomen as 7.7%(95% Cl = 7.2–8.2%), 7.8 %(95% Cl = 7.1–8.5%) and 3.6%(95% Cl = 3.0–4.3%), respectively (130). In a retrospective study of COVID-19 patients, when comparing digestive-only, respiratory-only, and digestive and respiratory groups, stool RNA was positive in 60, 14.3, and 80% patients, respectively. It took a long time to clear the virus in a patient with positive viral RNA in stool compared to those with a negative test (44.2 vs. 33.7 days, *P* = 0.003). The diarrhea duration in COVID-19 can last up to 14 days, with an average duration of 5.4 ± 3.1 days (131).

Recently more symptoms are being reported like loss of smell and taste sensation (132). A meta-analysis of 27 studies showed a pooled prevalence of loss of smell and taste in these patients to be 41.47% (95% Cl 3.13–31.03%) and 35.04% (95% Cl 22.03–49.26%), respectively (133). Both of these symptoms presented in patients on average on the fourth day after initial symptoms of the disease, but 13–15.5% of patients had a loss of smell and taste sensation as the first symptom (134, 135).

COVID-19 is a prothrombotic state leading to both microvascular and macrovascular thromboembolic events in pulmonary and extrapulmonary organs (136). Venous thromboembolism, particularly pulmonary embolism, is the most common coagulopathic manifestation in COVID patients (137). Several proposed mechanisms for thrombosis in COVID-19 patients include angiotensin-converting enzyme-2 receptor-mediated endothelial damage leading to cytokine storm, intussusceptive angiogenesis, and macrophage activation syndrome leading to activation of the coagulation cascade (136–139). The incidence of thrombotic events in COVID patients is 7.7–49% in various retrospective and prospective studies (140–144).

About 47% of patients with COVID-19 were without any comorbidities (129). A systematic review of thirty-one articles with comorbidity-specific data showed that diabetes mellitus (8.55%), cardiovascular/cerebrovascular disease (8.03%), respiratory disease (6.19%), and hypertension as most prevalent comorbidities in COVID-19 positive patients (145). Another systematic review of ten studies found 33.9% of the overall prevalence of obesity in hospitalized patients with COVID-19. Patients with obesity (defined by BMP> = 25) had higher odds of poor outcomes compared to a better outcome with a pooled odds ratio of 1.88 (95% CL:1.25–2.80, *p* = 0.002) (146). A meta-analysis of 212 studies showed that patients with severe disease were much older than (60.4 years, 95% Cl = 57.8–63.1) than patients with non-severe disease (44.6 years, 95% Cl = 42.8–46.3), *p* < 0.0001. It also showed that more men were in severe group (60.8%, 95% Cl = 57.2–64.2) compared to the non-severe group (47.6, 95% Cl = 44.9–50.4%), *p* < 0.0001 (147).

### Diagnosis

#### Laboratory Diagnosis

Table 4 outlines the case definitions used by WHO for surveillance. It is crucial to make a rapid and accurate diagnosis, especially in the current pandemic situation. The RT-PCR, real-time RT-PCR (rRT-PCR), and reverse transcription loop-mediated isothermal amplification (RT-LAMP) are currently available diagnostic tests, which detects unique sequences of virus RNA by nucleic acid amplification test (NAAT) to make the diagnosis (Table 1). RT-PCR assays target the RNA-dependent RNA polymerase (RdRp)/helicase (Hel), spike (S), and nucleocapsid (N) genes of SARS-CoV-2 (149, 150). NAAT test can be done on upper respiratory specimens (nasopharyngeal and oropharyngeal swab or wash), lower respiratory specimens [sputum (if produced) and/or endotracheal aspirate or bronchoalveolar lavage], blood and stool samples (149). Although upper and lower respiratory specimens are most commonly used for the test, a study of 73 hospitalized patients with COVID-19, stool SARS-COV-2 RNA test was positive for 53.4% patients, and in 23% cases stool test remained positive even after a negative respiratory test (125).

**Table 4:** WHO case definitions for surveillance and last updated on March 20th, 2020.

**WHO Case Definitions for Surveillance March 20, 2020**	
Suspected case	•A patient with acute respiratory illness, AND a history of travel to or residence in a location reporting community transmission of COVID-19 during the 14 days prior to symptom onset OR •A patient with any acute respiratory illness AND having been in contact with a confirmed or probable COVID-19 case (see definition of contact) in the last 14 days prior to symptom onset OR •A patient with severe acute respiratory illness (fever and at least one sign/symptom of respiratory disease, e.g., cough, shortness of breath; AND requiring hospitalization) AND in the absence of an alternative diagnosis that fully explains the clinical presentation
Probable case	•A suspect case for whom testing for the SARS-CoV-2 is inconclusive OR •A suspect case for whom testing could not be performed for any reason
Confirmed case	A person with laboratory confirmation of COVID-19, irrespective of clinical signs and symptoms

*It includes the definition for the suspected, probable, and confirmed case, and these definitions may need to adapt further based on epidemiological situations (148)*.

### Case Definition

Defining the COVID-19 case is essential not only at the individual level but also from the public health perspective. WHO gave guidelines for defining a case as a laboratory-confirmed case of COVID-19 in the area with no known COVID-19 virus circulation and also in the area with established virus circulation.

**a**. *In an area with no known COVID-19 virus exposure*

- A case considered as laboratory-confirmed by NAAT: If a patient has positive NAAT result for at least two different targets on the COVID-19 virus genome, of which at least one target is preferably specific for COVID-19 virus using a validated assay; (OR)
- One positive NAAT result for the presence of beta coronavirus and COVID-19 virus further identified by sequencing the partial or whole genome of the virus as long as the sequence target is larger or different from the amplicon probed in the NAAT assay used (149).

**b**. *In an area with established COVID-19 virus exposure*

A screening by rRT-PCR using a single discriminatory target can be sufficient to consider a case laboratory-confirmed by NAAT.

One or more negative tests do not rule out the possibility in a patient with a high suspicion of COVID-19. Some of the factors which could explain at least in part for negative results include poor quality of the specimen, specimen not handled appropriately, collected very early or late in infection, use of only upper respiratory tract sample. In these cases, a sample should be collected and tested again, including a lower respiratory tract sample, if possible (149). Serological tests can be used to identify asymptomatic cases, diagnosis, and study the extent of outbreak retrospectively. In a patient with a negative NAAT and high suspicion for COVID-19, paired serum samples (in the acute and convalescent-phase) can be used to make the diagnosis (149). In a study of 285 patients with COVID-19, Immunoglobulin-G (IgG) and IgM levels were checked for patients. Hundred percentage of patients had positive virus-specific IgG within 17–19 days after symptom onset, and 94.1% of patients had IgM positive within 20–22 days after symptom onset. IgM and IgG levels plateaued within 6 days after seroconversion (151). Viral cultures are not recommended as a routine diagnostic test (149).

There are non-specific laboratory abnormalities observed in patients with COVID-19 infection. The most common laboratory findings include lymphopenia, elevated C-reactive Protein (CRP), elevated aspartate aminotransferase, hypoalbuminemia, elevated procalcitonin level, elevated D-dimer and erythrocyte sedimentation rate (ESR) (152–154). Serum levels of pro-inflammatory cytokines (interleukins, MCP1, MIP1A, MIP1BTNFα, IFNγ, IP10, and MCP1) were found to be elevated in patients with COVID-19. Furthermore, a higher concentration of GCSF, IP10, MCP1, MIP1A, and TNFα were noted in critically ill individuals requiring treatment in the intensive care unit (155). Along with the clinical presentation of COVID-19, elevated serum CRP may be used as a marker for the presence and severity of the disease (152).

#### Radiographic Diagnosis

Chest CT scan is the primary screening imaging modality for COVID-19. Ground glass opacities (GGO), consolidation, paving stone sign (finding ground-glass opacities with lobular interval thickening and interlobular interval lines), pleural thickening, and vascular thickening, and fibrinous lesions are common CT chest findings seen in a patient with COVID-19 (156, 157). Pleural effusion, pericardial effusion, and lymphadenopathy are rarely observed on CT scans in these patients (157). In a study comparing CT scan findings of COVID-19 and non-COVID pneumonia were GGOs (100% vs. 90.0%), mixed GGO (63.6% vs. 72.7%) and consolidation (54.5% vs. 77.3%), respectively. Pulmonary opacifications were more common in the peripheral area in COVID-19 than non-COVID-19 groups (100% vs. 31.8%, *p* = 0.05) (158).

Although NAAT is a gold-standard test for COVID-19 diagnosis due to high specificity, its sensitivity is 30–50%. Expectedly, diagnosis can be falsely missed if NAAT is the only test used for diagnosis. Patients with epidemiological features and positive CT scan findings should be isolated, and the NAAT test to be repeated (153, 156). COVID-19 group had ground-glass opacity (GGO) or GGO with consolidation more frequently, whereas the non-COVID-19 pneumonia group has consolidation as a common finding on CT scan (*P* < 0.05) (153). Therefore, patients should be isolated and rRT-PCR to be repeated in case there is a high suspicion of COVID-19 on CT imaging but a negative initial rRT-PCR test.

### Treatment

The mainstay treatment for COVID-19 is supportive management, with oxygen and mechanical ventilation, if needed (159). Empiric antibiotics have been used to prevent superimposed infections (160). FDA gave emergency use authorization for Remdesivir on May 1st, 2020, and there are no other FDA-approved medications available for COVID-19 (159–162). WHO announced the launch of an international clinical trial called SOLIDARITY trial on Match 18th, 2020, to help find an effective treatment of COVID-19. This trial will compare various options against the standard of care to assess the efficacy of these treatments. It will also add other drugs based on emerging evidence. This trial started to compare four treatment options (Remdesivir; Lopinavir/Ritonavir; Lopinavir/Ritonavir with Interferon beta-1a; and Chloroquine or Hydroxychloroquine) to the standard of care and study efficacy of these treatments. Hydroxychloroquine vs. standard of care and lopinavir/ritonavir vs. standard of care trials were discontinued on July 4th, 2020 by WHO based on the evidence presented at WHO Summit on COVID-19 research and innovation on July 1st and 2nd 2020. Overall, over 100 countries are participating in this trial (163).

The following treatments are currently being used for COVID-19 due to the effects seen *in vitro*.

#### Protease Inhibitors

##### Lopinavir-Ritonavir

For the treatment of COVID-19, the NIH panel recommends against the use of lopinavir/ritonavir and other HIV protease inhibitors unless it is for a clinical trial (159). Lopinavir is a highly potent inhibitor of the HIV protease essential for intracellular HIV assembly, and its half-life increases when combined with ritonavir via cytochrome P450 inhibition (161, 164). Lopinavir/ritonavir inhibits SARS-CoV-2 3CLpro *in-vitro* and thus suppress the cleavage of polyproteins into multiple functional proteins like RNA polymerase and a helicase (159, 160). In a randomized, controlled, open-label trial of 199 hospitalized patients with SARS-CoV-2 infection, patients were randomized in a 1:1 ratio to either lopinavir-ritonavir (400 and 100 mg, respectively) twice a day for 14 days along with standard care, or standard care alone. There was no difference in time for clinical improvement, mortality at 28 days, and detectable viral load was seen in the lopinavir-ritonavir group compared to standard treatment. Severe adverse events were seen more commonly in the standard treatment group, but the lopinavir-ritonavir group showed more gastrointestinal (nausea, vomiting, and diarrhea) adverse effects (165).

##### Darunavir/Cobicistat

Darunavir/Cobicistat is another protease inhibitor used in HIV patients. No clinical trials have been conducted yet in the US. A single unpublished trial from China showed that it was not effective in COVID-19 treatment as darunavir has low affinity for coronavirus protease (159).

#### Remdesivir

It is an analog of adenosine, nucleotide prodrug, which inhibits viral RNA replication by interfering with the activity of viral RNA-dependent RNA polymerase (RdRp) (150, 161). It has shown activity against Ebola in rhesus monkeys, and other RNA viruses, including arenaviruses and coronaviruses (161, 164). Remdesivir has inhibitory activity against SARS-CoV-2 infection at EC90 of 1.76 μM, in *in-vivo* non-human primate models (164). It also has inhibitory effects against SARS-CoV-2 infection of Human Liver cells, Huh-7 cells (160, 164). In a study of 53 patients who received at least one dose of remdesivir on a compassionate-use basis, clinical improvement was noticed in 68% (36/53) patients. 57% (17/30) patients were extubated who were receiving mechanical ventilation. The overall mortality rate was 13%, but it was higher (18%) in patients receiving mechanical ventilation (166).

A preliminary update from a randomized controlled trial involving 1,063 patients called Adaptive COVID-19 Treatment Trial (ACTT) sponsored by the National Institute of Allergy and Infectious Diseases (NIAID) indicates that patient who received remdesivir showed a 31% faster time to recovery than the placebo group (*p* < 0.001). It also suggested a lower mortality rate of 8% in the remdesivir group compared to 11.6% in the placebo group but did not reach statistical significance (*p* = 0.059) (167, 168). FDA gave emergency use authorization for Remdesivir use on May 1st, 2020, after preliminary results from the ACTT trial. Multiple clinical trials are in development to study remdesivir use in COVID-19 patients (169).

#### Chloroquine and Hydroxychloroquine

NIH panel recommends against the use of chloroquine or hydroxychloroquine for the treatment of COVID-19 in hospitalized patients. NIH panel also recommends against chloroquine or hydroxychloroquine for COVID-19 treatment in non-hospitalized patients, except in the context of a clinical trial. NIH panel also recommends against the use of hydroxychloroquine with azithromycin for COVID-19 treatment, except in the context of a clinical trial (170). Chloroquine and Hydroxychloroquine are immunomodulatory drugs that inhibit terminal phosphorylation of ACE2 and elevate pH in endosomes involved in virus cell entry. Hydroxychloroquine metabolizes into chloroquine *in-vivo* and may have lower adverse effects than chloroquine (159, 164).

Hydroxychloroquine was more potent than chloroquine *in-vitro* in SARS-CoV-2 infected Vero cells using physiologically-based pharmacokinetic (PBPK) models. This model also recommended an oral loading dose of 400 mg twice daily on day 1, followed by an oral maintenance dose of 200 mg twice daily for 4 days of hydroxychloroquine for patients with SARS-CoV-2 (171). For chloroquine, a dose of 500 mg is needed to achieve an EC90 value of 6.90 μM in Vero E6 cells (172). In a study conducted in China, 22 patients were randomized into two groups with one treated with chloroquine 500 mg orally twice daily for 10 days, and others treated with Lopinavir/Ritonavir 400/100 mg orally twice daily for 10 days. On day 10, 90% of patients in the Chloroquine group were SARS-CoV-2 RT-PCR negative compared to 75% in Lopinavir/Ritonavir group. CT scan improvement was 100% in the Chloroquine group and 75% in Lopinavir/Ritonavir group (173). In a randomized controlled study of 62 patients with two parallel groups with one assigned to receive 5 days of Hydroxychloroquine (400 mg/day) along with standard treatment and other assigned to control group receiving standard treatment, 80.6% of patients in the Hydroxychloroquine (HCQ) group compared to 54.8% in the control group showed improvement in pneumonia on CT imaging. HCQ group had 2.2 days vs. 3.2 days of mean duration fever and 2.0 days vs. 3.1 days of cough compared to the control group (174).

An observational study from France of 80 confirmed COVID-19 patients who received a combination of HCQ and azithromycin for at least 3 days and then followed for at least 6 days showed that the majority (81.3%) of patients were discharged from the unit as they had a favorable outcome. Rapid fall in nasopharyngeal viral load was noticed with 83% negative on Day 7 and 100% negative on Day 12 (175). Eighty-four COVID-19 positive patients were given a combination of HCQ and azithromycin as treatment. Eighteen percentage of these patients had an increase in QTc interval by 40 to 60 ms, and another 12% had an increase in Qtc by >60 ms. Acute renal failure (OR 19.45, 95% CI, 2.06–183.88, *P* = 0.01) was a strong predictor of extreme QTc prolongation instead of baseline QTc level (176).

#### Convalescent Plasma

NIH panel states that there is insufficient data to recommend either for or against the use of convalescent plasma or hyperimmune immunoglobulin for the treatment of COVID-19. Convalescent plasma has been used in the past for the treatment of various diseases, including SARS. In the United States, FDA had issued guidance for the use of convalescent plasma collected from individuals who have recovered from COVID-19 (COVID-19 convalescent plasma) for administration to a patient with COVID-19 and investigational studies during the public health emergency (177). A case series of 5 patients with laboratory-confirmed COVID-19 and ARDS received convalescent plasma infusion. In these patients, SOFA score decreased, PaO_2_/FiO_2_ increased, and viral load deceased and became negative within 12 days after transfusion. The ARDS resolved in 4 patients at 12 days after transfusion (178). Clinical trials are in development regarding the evaluation of the use of both convalescent plasma and SARS-CoV-2 IVIG to treat COVID-19 (179).

#### Antibodies

The Spike protein of CoV is a primary inducer of neutralizing antibodies. Cross-reactivity of the anti-SARS-CoV-1 antibody was checked with SARS-CoV-2 spike protein due to the similarity between the receptor-binding domain (RBD) in SARS-CoV-1 and SARS-COV-2. SARS-CoV-1 specific human monoclonal antibody CR3022 binds to SARS-CoV-2 RBD very strongly. A similar affinity was not seen with other SARS-CoV-1 RBD-directed antibodies 230, m396, and 80R. Given the above information, CR3022 can be a potential candidate for the treatment of COVID-19 infection (180).

#### Interleukins Inhibitors and JAK-Inhibitors

NIH Panel recommends against the use of Janus kinase (JAK) inhibitors (e.g., baricitinib) to treat COVID-19 unless it is for a clinical trial. There is insufficient data in favor of or against the use of Interleukin-1 inhibitors (e.g., anakinra) and IL-6 inhibitors (e.g., sarilumab, siltuximab, or tocilizumab)in the treatment of COVID-19. Interleukin inhibitors are therapies directed against the inflammatory cytokines or other parts of the innate immune response. It is proposed that significant tissue damage, including in lungs and other organs, is caused by exacerbated immune response and cytokine release (181). Interleukin-1 is a pro-inflammatory cytokine that induced IL-6 in macrophages and monocytes. It is elevated in patients with COVID-19, and other conditions, such as macrophage activation syndrome (MAS), severe chimeric antigen receptor T-cell (CAR-T) mediated cytokine release syndrome (CRS). Janus kinase (JAK) enzymes regulate signal transduction in immune cells (159). Interleukin inhibitors are thought to act by suppressing cytokine processes, which causes tissue damage (159, 181). Similarly, the JAK inhibitor can block the cytokine release. Thus, IL-1 and IL-6 blockades and JAK inhibition proposed a potential treatment option for patients with COVID-19 infection (159).

A phase 2/3 open-label, randomized parallel-group, three arms, multicenter study is underway in Italy to assess the efficacy and safety of intravenous Administrations of Emapalumab, an Anti-interferon Gamma (Anti-IFNγ) Monoclonal Antibody, and Anakinra, and Interleukin-1(IL-1) Receptor Antagonist, vs. Standard of Care, in Reducing Hyper-inflammation and Respiratory Distress in Patients With SARS-CoV-2 Infection. It was started in April with an estimated date of completion in Sept 2020 [141]. In a retrospective study conducted in China with 15 patients, Tocilizumab (TCZ), a monoclonal antibody against IL-6 was given to all patients. Eight patients received methylprednisolone along with TCZ. C-reactive protein (CRP) and IL-6 levels were checked before and after TCZ therapy. CRP level decreased significantly after TCZ therapy, dropped from 126.9 (10.7–257.9) to 11.2 (0.02–113.7) mg/L (*P* < 0.01). However, in four critically patients who received only one dose of TCZ, three of them died, and CRP did not return to normal within a week. IL-6 level spiked first before decreasing after receiving TCZ. Again, all four critically patients had a persistent increase in IL-6 even after getting TCZ. Given the above results, repeated doses might improve the condition in critically ill patients. IL-6 can be used to know the severity and prognosis of the disease. Since it was a small study, the results should be interpreted with caution (182).

#### Interferons

NIH panel recommends against the use of interferons for the treatment of COVID-19, except in the context of a clinical trial as there are no clinical trials and no proven benefits of interferons in other coronavirus infection and potential adverse effects outweigh benefits (159).

#### Corticosteroids

Both WHO and NIH panels recommend using systemic corticosteroids for patients with critical (mechanically ventilated patient) and severe (requiring supplemental oxygen) COVID-19 disease. Whereas, WHO and NIH panel recommends against corticosteroids in patients with non-severe (not requiring supplemental oxygen) COVID-19 disease (170, 183). These recommendations are based on a preliminary report from the Randomized Evaluation of COVID-19 Therapy (RECOVERY) trial. In this trial, 2104 patients were assigned to receive dexamethasone (6 mg once daily) oral or intravenous for up to 10 days and 4,321 to receive usual care alone. Dexamethasone group found to have lower mortality at 28 days after randomization than the usual care group with reported deaths 482/2,104 patients (22.9%) and 1,110/4,321 patients (25.7%), respectively (age-adjusted rate ratio, 0.83; 95% CI, 0.75–0.93; *P* < 0.001). Furthermore, the incidence of death was lower in the dexamethasone group compared to usual care group in patients on mechanical ventilation (29.3% vs. 41.4%; rate ratio, 0.64; 95% CI, 0.51–0.81) and one receiving supplemental oxygenation (23.3% vs. 26.2%; rate ratio, 0.82; 95% CI, 0.72–0.94) but no clear effects were seen in patients without any supplemental oxygen (17.8% vs. 14.0%; rate ratio, 1.19; 95% CI, 0.91–1.55) (184). In a systemic review and meta-analysis, 23 randomized trials reported mortality and showed lower mortality in the group randomized to glucocorticoids (odds ratio 0.87, 95% credible interval 0.77 to 0.98; risk difference 31 fewer per 1,000, 95% credible interval 55 fewer to 5 fewer; moderate certainty) than standard care (185).

#### Anticoagulation

Given the risk of thrombotic events in patients with COVID-19, the American Society of Hematology and the International Society on Thrombosis and Hemostasis recommends thromboprophylaxis with antithrombotic agents in all hospitalized COVID-19 patients unless there are contradictions (186, 187). Various societies like the American College of Chest Physician, American College of Cardiology, Anticoagulation Forum, American Society of Hematology, and CDC recommends against using therapeutic anticoagulation unless there is a confirmed or high suspicion of thrombotic events and other indications of anticoagulation like atrial fibrillation, mechanical cardiac valves and secondary venous thromboprophylaxis (170, 187–190). A single-center, open-labeled randomized controlled study of 20 COVID-19 positive patients requiring mechanical ventilation were randomized to either therapeutic or prophylactic dose of enoxaparin. Patients in the therapeutic enoxaparin group showed a significant increase of PaO_2_/FiO_2_ ratio of 163, 209, and 261 at baseline, after seven days and 14 days, respectively (*p* = 0.0004). Whereas, in the prophylactic enoxaparin group, no statistically significant difference in PaO_2_/FiO_2_ was noticed over time. Similarly, the therapeutic enoxaparin group (15 days [interquartile range, IQR 6–16)] had higher ventilator-free days compared to the prophylactic enoxaparin group (0 days [IQR 0–11)], *p* = 0.028. No difference was found in all-cause mortality and in-hospital mortality between the two groups. Although this study shows that therapeutic enoxaparin improves gas exchange and ventilator-free day in severe COVID-19 patients, further large randomized clinical trials are needed as it was a single-center study with a small sample (191).

### Prevention

#### Vaccines

The genetic sequence of SARS-CoV-2 was revealed on 11 January 2020. It provides the basis of further studies to develop treatment and vaccines against SARS-CoV-2. Based on vaccine development pathways for other coronaviruses like MERS and SARS, pathways like nucleic acid, subunit vaccines, inactivated or live attenuated vaccines, and virus vector-based, are being investigated., The majority of vaccines in development are targeting S protein (150, 192). WHO is coordinating and directing global efforts to develop and evaluate vaccine candidates through global collaboration, development of robust methods, accelerating progress and avoiding duplication of research efforts, and coordinating efforts to rapidly and simultaneously assessing many vaccines (193) (Table 5). As of July 7th, 2020, there are 21 vaccine candidates in clinical evaluation and 139 candidates in the preclinical evaluation as per WHO (193).

**Table 5:** Eight candidate vaccines in clinical evaluation- obtained from WHO DRAFT landscape of COVID-19 candidate vaccines−11 May 2020 (193).

**Platform**	**Type of candidate vaccine**	**Developer**	**Current stage**
Non-replicating viral vector	Adenovirus Type 5 vector	CanSino Biological Inc./Beijing Institute of Biotechnology	Phase 2 ChiCTR2000031781 Phase 1 ChiCTR2000030906
Inactivated	Inactivated	Wuhan Institute of Biological Products/Sinopharm	Phase1/2 ChiCTR2000031809
Inactivated	Inactivated	Beijing Institute of Biological Products/Sinopharm	Phase 1/2 ChiCTR2000032459
Inactivated	Inactivated + alum	Sinovac	Phase 3 NCT04456595 Phase 1/2 NCT04352608 NCT04383574
DNA	DNA plasmid vaccine	Candila Healthcare Limited	Phase 1/2 CTR1/2020/07/026352 (not yet recruiting)
Non-replicating viral Vector	ChAdOx1-S	University of Oxford/AstraZeneca	Phase 3 ISRCTN89951424 Phase 2b/3 2020-001228-32 Phase 1/2 PACTR202006922165132 2020-001072-15
RNA	3 LNP-mRNAs	BioNTech/Fosun Pharma/Pfizer	Phase 1/2 2020-001038-36 NCT04368728
DNA	DNA plasmid vaccine with electroporation	Inovio Pharmaceuticals	Phase 1/2 NCT04447781 NCT04336410
Protein subunit	Full length recombinant SARS CoV-2 glycoprotein nanoparticle vaccine adjuvanted with Matrix M	Novavax	Phase 1/2 NCT04368988
DNA	DNA Vaccine (GX-19)	Genexine Consortium	Phase 1 NCT04445389
DNA	DNA plasmid vaccine +Adjuvant	Osaka University/AnGes/Takara Bio	Phase 1 JapicCTI-205328
Inactivated	Inactivated	Institute of Medical Biology, Chinese Academy of Medical Sciences	Phase 1 NCT04412538
Non-replicating viral vector	Adeno-based	Gamaleya Research Institute	Phase 1 NCT04436471 NCT04437875
Protein subunit	Native like trimeric subunit Spike Protein vaccine	Clover Biopharmaceuticals Inc./GSK/Dynavax	Phase 1 NCT04405908
Protein subunit	Adjuvanted recombinant protein (RBD-Dimer)	Anhui Zhifei Longcom Biopharmaceutical/Institute of Microbiology, Chinese Academy of Sciences	Phase 1 NCT04445194
Protein subunit	Recombinant spike protein with Advax^TM^ adjuvant	Vaxine Pty Ltd/Medytox	Phase 1 NCT04453852
RNA	LNP-nCOVsaRNA	Imperial College London	Phase 1 ISRCTN17072692
RNA	mRNA	Curevac	Phase 1 NCT04449276
RNA	mRNA	People's Liberation Army (PLA) Academy of Military Sciences/Walvax Biotech	Phase 1 ChiCTR2000034112
VLP	Plant-derived VLP	Medicago Inc./Universite Laval	Phase 1 NCT04450004 (not yet recruiting)
RNA	LNP-encapsulated mRNA	Moderna/NIAID	Phase 2 NCT04405076

*Reproduced and published under Creative Commons Attribution-NonCommercial-ShareAlike 3.0 Intergovernmental Organization License: CC BY-NC-SA 3.0 IGO*.

## Conclusion

Over the last 20 years, three coronaviruses have been transmitted from animals to humans who have resulted in epidemic or pandemic. SARS-CoV-1 led to the first epidemic of the twenty first century crippling the healthcare system of the affected countries. A WHO-led global response to this disease through a virtual network of laboratories and health systems worldwide helped limit its spread. There have been no new cases since 2004, but it remains a potential threat in the future. MERS emerged in 2012 and still exists in dromedary camels, and it has the potential to infect people who have close contact with them. The majority of human cases of MERS occurred due to human-to-human transmission in the healthcare setting. Hence, early recognition of a case and implementation of internationally recommended infection control measures are needed to prevent healthcare facility associated outbreaks. COVID-19 is the latest deadly respiratory illness that is believed to have originated in a live animal market in China. Its rapid spread has become a pandemic and continues to threaten the healthcare system and the world's economy. Stringent public health measures such as social distancing, contact tracing, testing, quarantines, and travel restrictions are of paramount importance to control the spread. Scientists are working to find medications to treat the disease and to develop a vaccine. Multiple vaccines are currently in various trials. As now, there is no specific treatment or vaccine for COVID-19; therefore, prevention measures are critical. These zoonotic infections are the consequences of urbanization, agricultural work, and other human activities. There are currently no specific antiviral medications for SARS, MERS, or COVID-19. There are still knowledge gaps in understanding the pathophysiology, viral kinetics, and duration of viral shedding of COVID-19, which is a significant limitation in developing effective treatment and vaccines. Moreover, there is a significant lack of knowledge about natural history and clinical courses in special populations like pregnant patients and children. Therefore, well-coordinated international collaborative research needs to be done on the pathogenesis of human coronaviruses, which is needed to develop treatment and preventative measures against coronaviruses.

## Author Contributions

HG: conception and design. MG: formal analysis. AP, MG, and HG: literature search. RM: first draft. All authors critical revision and editing and final approval.

## Conflict of Interest

The authors declare that the research was conducted in the absence of any commercial or financial relationships that could be construed as a potential conflict of interest.
